# A self-avoidance mechanism in patterning of the urinary collecting duct tree

**DOI:** 10.1186/s12861-014-0035-8

**Published:** 2014-09-10

**Authors:** Jamie A Davies, Peter Hohenstein, C-Hong Chang, Rachel Berry

**Affiliations:** 1 University of Edinburgh, Edinburgh EH8 9XB, UK; 2 The Roslin Institute, University of Edinburgh, Easter Bush Campus, Midlothian EH25 9RG, UK; 3 The Institute of Genetics and Molecular Medicine, Western General Hospital, Crewe Road, Edinburgh EH4 2XU, UK

**Keywords:** Adaptive self-organization, Branching morphogenesis, Ureteric bud, Kidney development, Metanephros, Signalling, Repulsion, Pathfinding, Navigation

## Abstract

**Background:**

Glandular organs require the development of a correctly patterned epithelial tree. These arise by iterative branching: early branches have a stereotyped anatomy, while subsequent branching is more flexible, branches spacing out to avoid entanglement. Previous studies have suggested different genetic programs are responsible for these two classes of branches.

**Results:**

Here, working with the urinary collecting duct tree of mouse kidneys, we show that the transition from the initial, stereotyped, wide branching to narrower later branching is independent from previous branching events but depends instead on the proximity of other branch tips. A simple computer model suggests that a repelling molecule secreted by branches can in principle generate a well-spaced tree that switches automatically from wide initial branch angles to narrower subsequent ones, and that co-cultured trees would distort their normal shapes rather than colliding. We confirm this collision-avoidance experimentally using organ cultures, and identify BMP7 as the repelling molecule.

**Conclusions:**

We propose that self-avoidance, an intrinsically error-correcting mechanism, may be an important patterning mechanism in collecting duct branching, operating along with already-known mesenchyme-derived paracrine factors.

## Background

Pattern formation in branching morphogenesis has been the subject of biological speculation since the beginning of embryology [1]. On the one hand, theoreticians have stressed that branched trees have a self-similar (fractal) nature that suggests a simple, repetitive mechanism of generation [2],[3]. On the other, anatomists have stressed that branching systems of different organs are easily distinguishable even in silhouette and that, even within the same organ, different generations of branching are distinct. In particular, the first branch events of an organ follow a stereotyped pattern different from subsequent branch events, a fact that has prompted the suggestion that the early branching events might be under the control of a special genetic program [4]. The competition between the general repetitive and the particular, sequential models has prompted much research into the molecular cell biology of branching morphogenesis [5].

**Figure 1:**
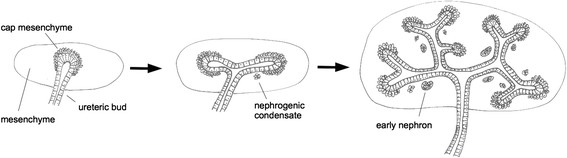
**Development of the renal ureteric bud/collecting duct system.** The ureteric bud begins as an unbranched epithelial tube that invades the metanephrogenic mesenchyme, and recruits cells of that mesenchyme to form a `cap¿ (this cap becomes the stem cell population that produces nephrons). The ureteric bud then bifurcates, and the cap splits along with it. As the branches of the ureteric bud grow, pieces of distal cap left behind differentiate to make first a nephrogenic condensate and then to become an epithelial early nephron. This process repeats to form a ureteric bud tree (the future collecting duct) and the nephrons that will later connect to it. A much more detailed, illustrated account of renal development can be found at www.gudmap.org.

Epithelial branching in developing mammalian organs is now known to be regulated by a large number of factors including mesenchyme-derived signalling molecules and extracellular matrix [6],[7]. Patterning of the tree is usually assumed to be achieved by spatial variation in the production or diffusion of paracrine factors [8] and, in chimeric organs, branching anatomy is controlled mainly by the origin of the mesenchyme [9]. These paracrine factors are undoubtedly important but both epithelial cell lines [10],[11] and intact epithelia [12] can undergo branching morphogenesis in 3D gels with these factors provided in free solution, with no mesenchymal cells present. The shape of the trees in these 3D gel systems is not completely identical to the organ concerned (it tends to extend in all directions, without the characteristic overall shape of a lung, kidney etc.) but they do consist of a tree with spreading branches. This implies that the epithelium must have its own basic tree-patterning system that causes branches to form and spread out, even when normally mesenchyme-derived factors are ubiquitous rather than patterned. Exploration of this idea using mammary epithelial cells in advanced cell culture systems [13] has suggested the possibility of patterning by autocrine secretion, by the epithelial cells, of an inhibitor of invasive activity. Here, we extend this idea, previously explored only in simple cell culture, to intact and growing collecting duct trees of developing kidneys growing in organ culture (Figure 1). We find evidence for an autocrine tree-patterning system that spreads branches out by mutual repulsion. The system can also account for the `special¿ anatomy of first branches without the need for any extra `special¿ mechanism. The system involves bone morphogenetic protein 7 (BMP7) signalling.

## Results

### First branch divergence is significantly greater than that of subsequent branches

In the branched epithelia of developing glandular organs such as kidney and lung, the first branch shows a divergence angle markedly different from divergence angles of subsequent branching events, at least once the branches have had time to elongate [14]. Throughout this report, we use `divergence angle¿ to refer to the relative directions at which branches lie after they have elongated and responded to any guidance cue present in the system. No claim is made or implied about the shape of a branch tip at the moment of bifurcation.

**Figure 2:**
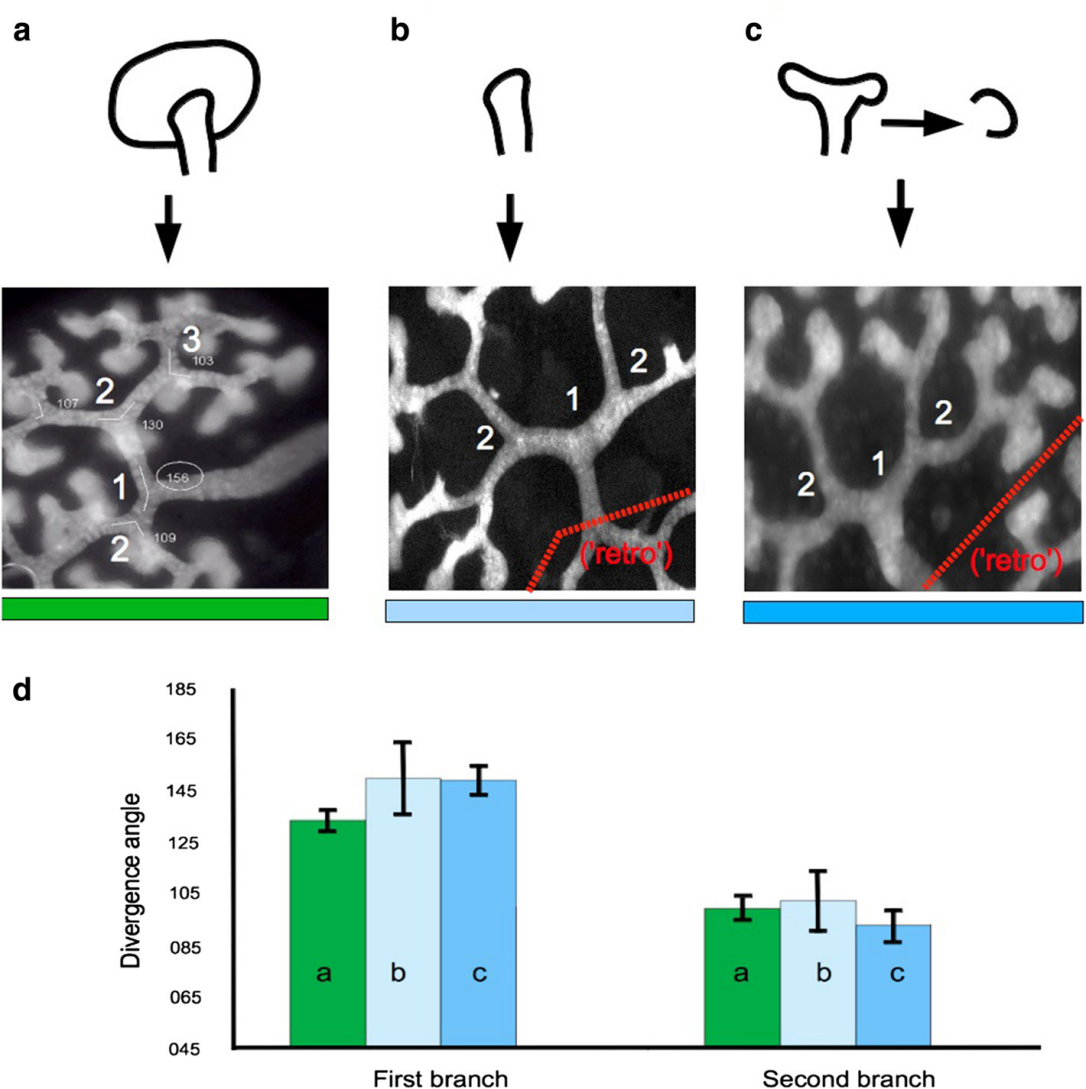
**Divergence angles of branching tubules are governed not by sequence but by the presence of other tips.** Normal kidneys cultured intact **(a)** show a wide angle of first branching (`1¿), and narrower second (`2¿) and subsequent branches. Real angles from this specimen are indicated on the figure and mean values can be seen in the green bars of Figure 1d. An unbranched ureteric tip cultured with its own mesenchyme **(b)** shows a similar wide-then-narrower pattern. An already-branched tip **(c)**, which would naturally go on to produce a narrow branch angle, begins by producing a wide angle characteristic of the first branch when it is cultured alone (with its own mesenchyme). In the images, the area behind the red dotted line, labelled `retro¿, is a branching system that develops from the bladder end of the cut ureteric bud, behaviour that has already been described [46]: data were not gathered from the `retro¿ branching system because its first branch occurred later then the normal ones, with very variable timing. **(d)** Shows branching angles quantitatively, a,b, and c referring to the culture methods shown in **(a)**, **(b)** and **(c)** and colours in the graph matching the colour bars under each micrograph: error bars represent standard error of the mean. In all cases, second branch angles differ from first branch angles with p?<?0.05 (p values are given in the main text).

Metanephric kidney rudiments cultured on filters supported by Trowell screens grow essentially `two-dimensionally¿. They are thick enough for intact ureteric bud/collecting duct and nephron tubules to form, but are too shallow for these tubules to elongate in any plane except parallel to the substrate. The culture system therefore reduces the problem of tree formation to two dimensions, making analysis and intervention easier. To determine whether the phenomenon of the `special¿ first divergence angle is seen in the two-dimensional system, we measured the divergence angles of first and second generation branches. The first branches (Figure 2a; quantitative information in Figure 2d) showed a mean divergence angle of 133° (n?=?33, ??=?21.3°) while the next generation of branches showed a mean divergence angle of 99.9° (n?=?34, ? =24.3°; p?=?5.7 × 10^?8^).

### Branch divergence is controlled by presence of other branches

The difference between first and subsequent divergence angles [14] might be explained by arguing that the first branch is made a special `early branch¿ mechanism, before control is handed to a routine branching programme [4]. An alternative hypothesis would be that the branching mechanism is the same for all branching events but the angles are controlled by the environment, specifically the presence of other bud tips. To test these two models, branch tips (epithelia and their associated mesenchyme) isolated from unbranched, or from already once-branched, ureteric buds were cultured and the divergence angles of their next-formed branches were measured. Isolated tips from *un*branched ureteric buds (Figure 2b) branched first with a wide divergence angle (mean?=?149°, ??=?20°) then diverged more acutely (mean?=?93°, ??=?19°, p?=?0.018). Tips from buds that had already branched once and were placed in isolation made another open initial divergence angle (128° ??=?11°) characteristic of a normal first branch (Figure 2c). Subsequent branch events were more acute, as expected for second branch events (82°, ??=?22°, p?=?1.8 × 10^?6^). These data (summarized quantitatively in Figure 2d) show that the change in divergence angle between first and later branching events is controlled by the presence or absence of another nearby tip.

### A simple, qualitative computer model for self-avoidance

The direction taken by new branches in all of the cases described above have one thing in common: the branches seem to maximize their separation from other nearby branches. We used computer modelling to test if a secreted repulsive factor could achieve such patterning. The word `model¿ is sometimes misunderstood: we emphasize that what we present here is not intended to be a formal description of a real kidney (far too little is known about real rate constants, diffusion constants etc. for such a thing to be possible), but is a simplified system in which ideas can be explored in principle and used to direct experimental confirmation. The model is intended just as an abstract thinking tool to identify promising lines of wet-lab experimentation, and the conclusions of this manuscript rest on the wet lab data, not the details of the model.

The model is of the cellular Potts type, in which the tissue is represented by a two-dimensional grid of locations, each of which has a few associated parameters such as concentration of a particular molecule, or occupation by part of a ureteric bud tree. The `tip¿ and `stalk¿ components of the tree are represented with distinct identities. Tree tubules are considered to be sources of a factor, *horrid*, that diffuses away from them. The concentration of *horrid* arising from any particular point of the tubule, measured at another location in the tissue, decreases exponentially with distance, as would happen for first order decay/loss of a molecule that is either short-lived or is lost to the bulk medium above or below the plane of the tissue. The total concentration at any one point in the tissue is taken as the sum of the contributions to that place from each part of the bud, with some random noise added. The model makes the simplifying assumption that he diffusion of *horrid* is rapid compared to the speed of growth of the tubules: this is justified by the observation that treating real cultured kidneys with even large proteins such as growth factors or antibodies can produce an immediate effect on subsequent development of their ureteric bud trees, demonstrating that protein diffusion in the system is rapid compared with tree growth. Making this assumption allows the concentration gradients to be calculated at each stage from current tree anatomy, with no need for history to be taken into account. The model begins with one or more unbranched stalks. The tip(s) of the stalk(s) and subsequent tree(s) bifurcate only when the local concentration of *horrid* is below a threshold, and the new tips are regarded as instantly making their own contribution to the *horrid* field (we make no claim that control of branch timing by an inhibitor is true of real ureteric buds: the model has to have some mechanism to create branch points every so often, and the choice to use the concentration of *horrid* was made to avoid cluttering the model with any extra arbitrary features such as time intervals). Each tip advances at a rate determined inversely by its local concentration of *horrid*, in the direction of lowest local *horrid* as measured in the immediate vicinity of the tip. Stalks are left behind by advancing tips, as a slime trail may be left behind by an advancing snail. Further details of the model, source code and movies of its output, can be found in the Supplementary Data (Additional file 1: Code S1, Additional file 2: Movie S1, Additional file 3: Movie S2a, Additional file 4: Movie S2b, Additional file 5: Movie S3, Additional file 6: Movie S4, Additional file 7: Spreadsheet S1, Additional file 8: Text S1 and Additional file 9: Text S2).

**Figure 3:**
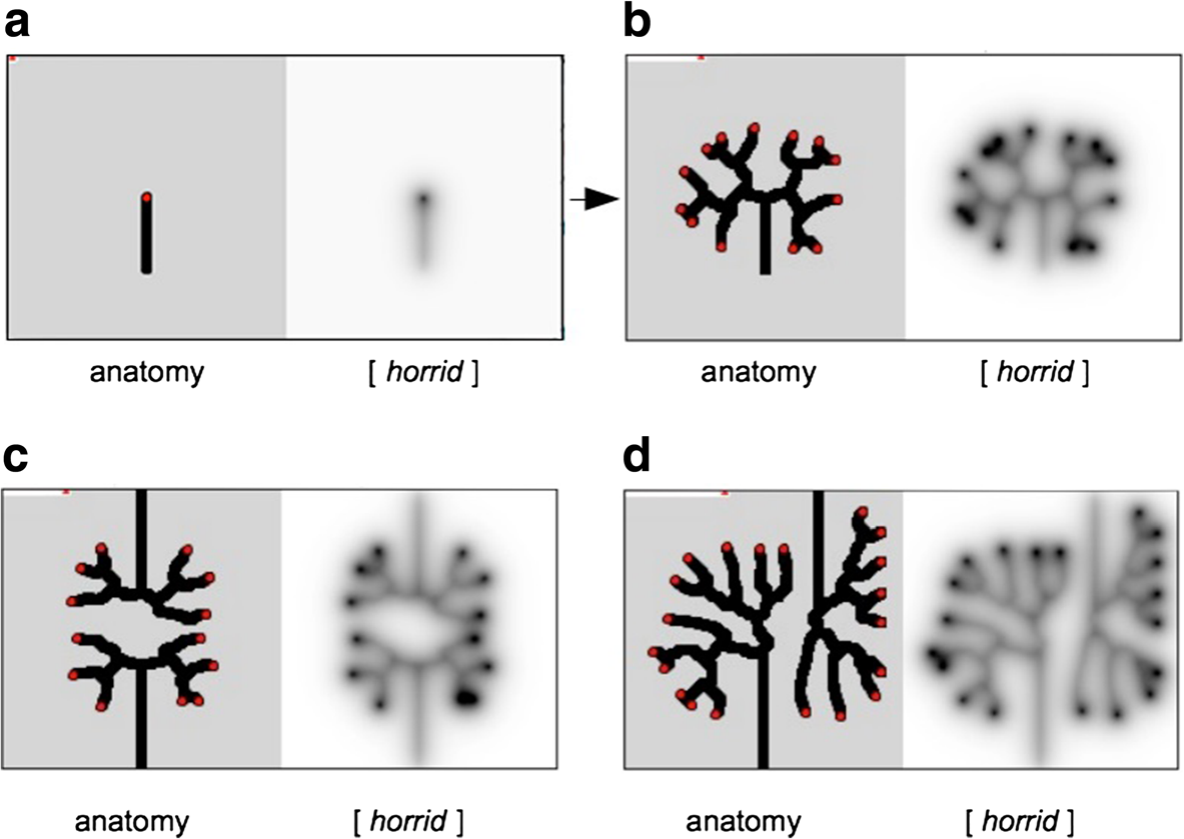
**Patterning of tubule trees by self-avoidance, in a simple model.** Beginning with an unbranched trunk **(a)**, secreting the repulsive factor *horrid*, the model produces a tree **(b)**, in which the first angle of branching is wide and subsequent angles narrower although this change is not written explicitly into the model, but emerges from self-avoidance. If two trunks are aimed at one another, either directly **(c)** or offset **(d)**, they each produce a tree that is distorted but that avoids collision with the other tree.

Beginning with an unbranched ureteric bud (Figure 3a), the model generates a realistic tree (Figure 3b), the branches spreading out automatically even in the presence of random noise. Notably, the angle of first branch is open (?150°) while the angles of subsequent branches are narrower (?95°). This narrowing of divergence angle is reminiscent of that seen in the real kidneys described above. It is important to note that no change of divergence angle was written directly into the simulation ¿ it emerged from the *un*changing rules.

Control of branch divergence by a secreted repulsive factor would be predicted to function also between buds from different trees. We tested this in the computer model. Beginning with two closely-spaced buds, either pointing at one another directly (Figure 3c) or offset (Figure 3d) the simulation produces trees that become distorted as mutual inhibition operates between branches belonging to different trees. This makes a prediction, testable in organ culture, that ureteric bud systems set up on collision courses will avoid contact even at the expense of making very distorted branch patterns.

### Ureteric bud trees of cultured kidneys avoid collision

**Figure 4:**
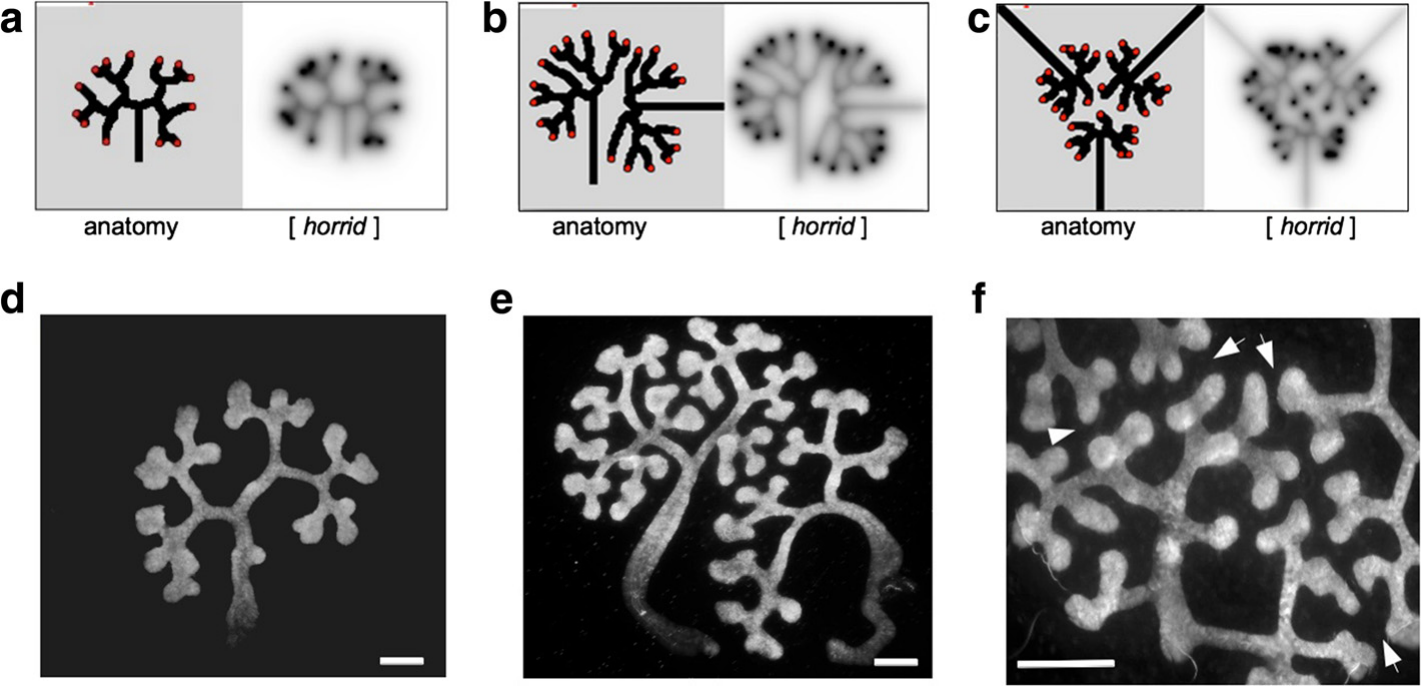
**Evidence for self-avoidance in the developing tubule trees of real cultured kidneys.** Single ureteric buds, isolated, surrounded by mesenchyme and cultured, generate reniform trees in both the model **(a)** and reality **(d)**. Pairs of ureteric buds cultured on collision courses with one another are predicted by the model to produce trees that are distorted but that avoid collision **(b)**: this does indeed happen in reality **(e)**. Where three ureteric buds are aimed towards one another, the model **(c)** and real cultures **(f)** generate straight `no-man¿s land¿, tip-free zones between them: these can be seen between the arrows in **(f)**. Additional file 11: Figure S4f shows the same image false-coloured to indicate more clearly which branches belong to which tree. Ureteric buds trees are stained with anti-calbindinD28^k^. Scale bars are 100 ?m.

When a single ureteric bud was isolated by microdissection, surrounded by metanephric mesenchyme and cultured alone as in the model shown in Figure 4a, it generated a typical reniform tree (Figure 4d). If two buds were cultured close to one another on a collision course in in the model shown in Figure 4b, their branch patterns were distorted from the usual outline so that collisions never occurred (Figure 4e), again in a manner broadly similar to the model. In this example, the number of branch points formed was the same (Additional file 10: Figure S4e) but some branches elongated far less than others, distorting the tree. It should be noted that the model (Figure 4b) shows fewer branch events in the region of apposition because the model uses inhibitor concentration to control both navigation and branching. The presence of bifurcations with short branches even in regions of close apposition in the real kidneys (Figure 4e, Additional file 10: Figure S4e) suggests that real ureteric buds do not base their decision of whether or not to branch on the local concentration of the inhibitor. Even when multiple ureteric buds were set up in close apposition, as in the model shown in Figure 4c, branches avoided contact or close approach, creating straight mutual boundaries between highly distorted trees (Figure 4f: a colour-coded version of this panel can be found as Additional file 11: Figure S4f). The anatomies of the model and the real kidney are not precisely identical (the model is, as stated earlier, just very simple abstraction that has only one signalling system in it): it is the prediction of collisions being avoided even at the expense of forming highly distorted trees that is relevant here.

**Figure 5:**
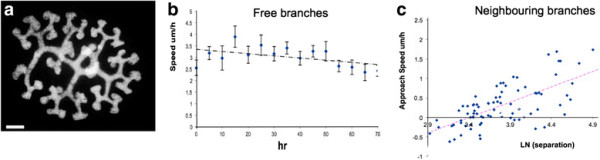
**Approaching branches slow and avoid contact even in intact cultured kidneys. (a)** shows an example frame from Additional file 2: Movie S1, a Hoxb7-cre x ROSA-eYFP kidney developing for a total of 6 days in culture (this construct causes the ureteric bud to fluoresce). **(b)** shows the speed of advance of branch tips that were not approaching other branches (the majority were of this type), at different times of culture. The mean speed is nearly constant over the culture, falling by only 12%. This is important for interpretation of panel **(c)**, which plots the closing speed of tips that are approaching one another against their separation. There is an inverse relationship between approach speed and log of distance, suggesting that the closing speed of branches decreases, even to a stop, as separation decreases (82 observations; R?=?0.67; significance?=?4.8x10^?12^). The change is is much larger than the 12% reduction of scalar speed in **(b)**, so cannot be accounted for simply by the culture ageing at the same time that tips approach other branches.

If repulsion between branches were relevant to normal development, one would expect that branches within a single kidney would slow down their growth speed as they approach other branches. To test this we measured the growth rate of kidneys with fluorescent ureteric buds through time-lapse imaging (Figure 5a). Over the 6-day period of culture, the rate of growth of cortical branches that are far from branches remained almost constant (Figure 5d), slowing only about 12% with age. When the speed of approach (closing speed) of adjacent cortical branch tips is plotted against their separation (Figure 5c) there is a significant negative correlation between closing speed and log of proximity (82 observations; R?=?0.67; p?=?4.8 × 10^?12^) before coming to a complete stop at a separation distance of 30 ?m. This confirms that self-avoidance is active during normal kidney development.

### Implication of signalling by the TGF?-superfamily, specifically BMP7, in collision avoidance

The ability of branching ureteric buds to avoid collisions even when cultured in close apposition was used as an assay to identify the signalling system involved. An obvious candidate signalling system for inhibiting epithelial advance is the TGF?-superfamily: cells from other branching systems such as mammary gland show a reduced motility from shaped wells in the presence of autocrine-secreted, accumulating TGF? [13],[15]. Furthermore, treatment of ureteric bud/collecting duct-derived cell lines with TGF? itself inhibits advance and branching of tubules in 3-dimensional collagen gel culture [16] and intact kidney rudiments [17].

**Figure 6:**
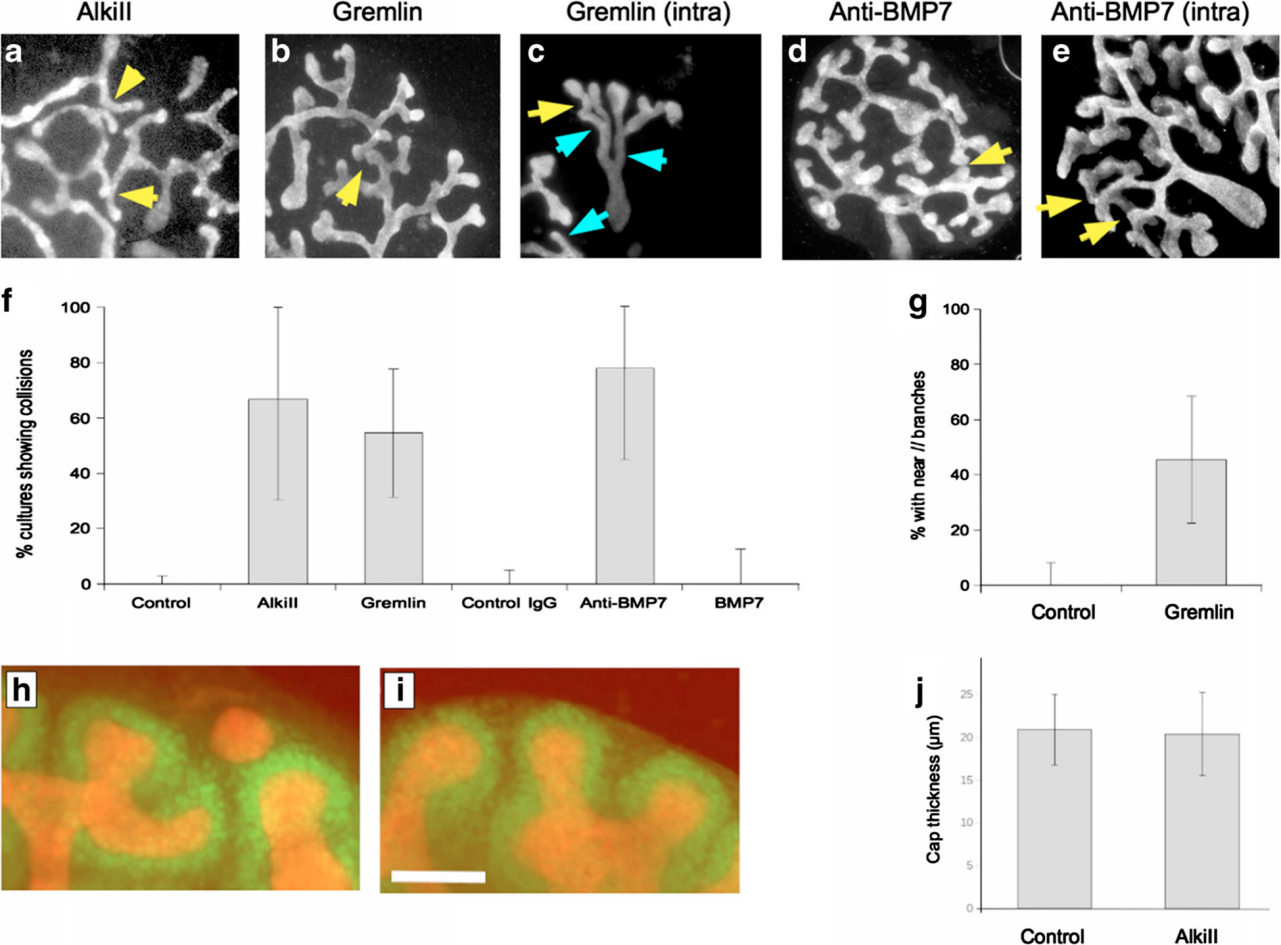
**Involvement of the BMP signalling in self-avoidance. (a)** In the presence of the inhibitor of TGF?-superfamily signalling, AlkiII, collision avoidance between co-cultured ureteric buds fails and branches make contact (arrowheads). In addition, branches become long and spindly. **(b)** Gremlin, a more specific inhibitor of signalling by BMPs, also causes collision avoidance between ureteric bud trees to fail, but the branch pattern is generally more normal than in AlkiII. **(c)** In some ureteric buds cultured in Gremlin, branches run almost parallel rather than diverging, and show inter-tree collisions. Anti-BMP7 also causes collision avoidance to fail between trees **(d)** and within trees **(e)**. **(f)** Shows the frequency of collisions quantitatively: none of the inhibitors causes collisions in every case, but each is significantly different from the controls, in which collisions were never seen (p values are in the main text). **(g)** Shows the incidence of parallel branches in Gremlin-treated buds. This was the only treatment to produce this effect reliably. **(h, i)** show the Six2-positive caps (green) over ureteric bud tips (red) in control and AlkiII-treated kidneys respectively; **(j)** shows quantitatively what is apparent visually from (h, i); the drug makes no detectable difference to the thickness of this cap, so the ability of AlkiII-treated tips to collide does not result from disappearance of a Six2+ `fender¿.

In the developing kidney, TGF? itself is absent from the early kidney, appearing in the ureteric bud/collecting duct system some time between E13.5 and E16 [18], 2 days after ureteric branching has begun, and falling away in the last days of renal development [19]. This makes it an unlikely candidate for patterning throughout tree growth. There are, however, many other members of the TGF?-superfamily and they converge on a core intracellular signalling pathway using Alk proteins [20]. Alk1,2,3,4,5,6,&7 are all inhibited by the drug AlkiII [2-(3-(6-methylpyridin-2-yl)-1H-pyrazol-4-yl)-1,5-naphthyridine] [21], which is therefore an inhibitor of signalling by Activin, BMP1-8, Gdf, Nodal and TGF?1,2&3. This drug was used to test whether a member of the TGF?-superfamily is involved in self-avoidance in the kidney. Treatment of apposed (`attempted collision¿) kidney cultures with 10 ?M AlkiII had two effects: it made branching less frequent along each tubule, and it resulted in branches from adjacent kidneys now being able to collide (Figure 6a). The difference between frequency of collisions (shown quantitatively in Figure 6f) in the presence (67%; n?=?9; CI^95%^ ±36%) or absence (0%; n?=?10; CI^95%^?=?5%) of this inhibitor was highly significant (p?=?0.0009). Normal ureteric bud tips are surrounded by a cap of Six2-positive cells (Figure 6h) that might, conceivably, act as a `fender¿ that normally prevents collisions. This is however still present and just as thick in the presence of AlkiII (Figure 6i,j), suggesting that AlkiII does not allow collisions by removing this fender. Also, each cap is about 20 ?m thick (Figure 6j) so, if collision prevention were to be mediated by the caps acting as fenders, closest approach would be expected to be 40 ?m, not the 30 ?m observed (see previous section).

**Figure 7:**
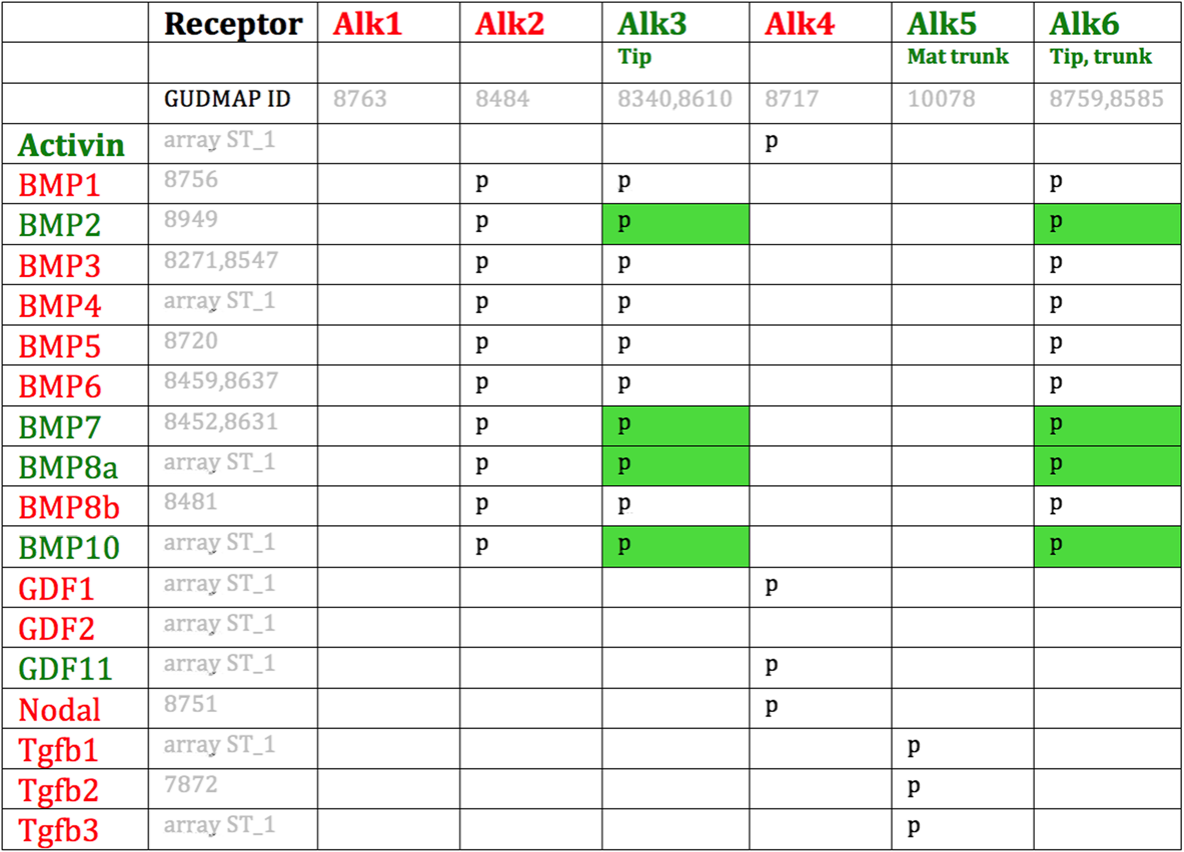
Expression of Alk receptors and their ligands, according to the GUDMAP database.

Whatever molecule signals through Alks to mediate self-avoidance, both the signalling molecule and the appropriate Alk must be expressed by growing branches. The pattern of Alk and ligand expression in the GUDMAP database of kidney development [22] is shown in (Figure 7) the only ligand/Alk combinations that satisfy the condition of co-expression are BMP 2,7,8a &10, signalling via Alk3/6. BMP signalling can be inhibited by Gremlin [23]: treatment of collision cultures with 5 ?g/ml Gremlin had a less dramatic effect on branching morphogenesis in general than did AlkiII, with tree morphology being basically normal rather than spindly, but there was still a significant failure of collision avoidance (Figure 6b,f): collisions occurred in 55% (n?=?22, CI^95%^?±?23%) of cultures compared to 0% (n?=?16; CI^95%^?±?8.3%) in controls (p?=?0.008). In cultures treated with Gremlin, some branches showed a very low divergence angle and ran almost parallel, occasionally colliding even in one tree (Figure 6c,g). The presence of almost-parallel branches of this type is very variable both within and between cultures but there is a clear difference between their frequency of occurrence in controls (0% of cultures; CI^95%^?±?8.3%) and Gremlin-treated cultures (45% of cultures; CI^95%^?±?23%; p?=?0.02). Of the four BMPs expressed in the ureteric bud, BMP7 is the only one that has strong expression from branch tips throughout renal development, even from the first branch events [24]. Inhibition of BMP7 function in culture, using a function-blocking antibody, results in collisions between adjacent trees (Figure 6d) and also collisions within the same tree to create occasional `loops¿ of collecting duct (Figure 6e): quantitatively there is again a clear difference between rates of collision in control IgG (0% of cultures; n?=?10; CI^95%^?±?5%) and anti-BMP7 (77% of cultures; n?=?9; CI^95%^?±?33%; p?=?0.0003).

**Figure 8:**
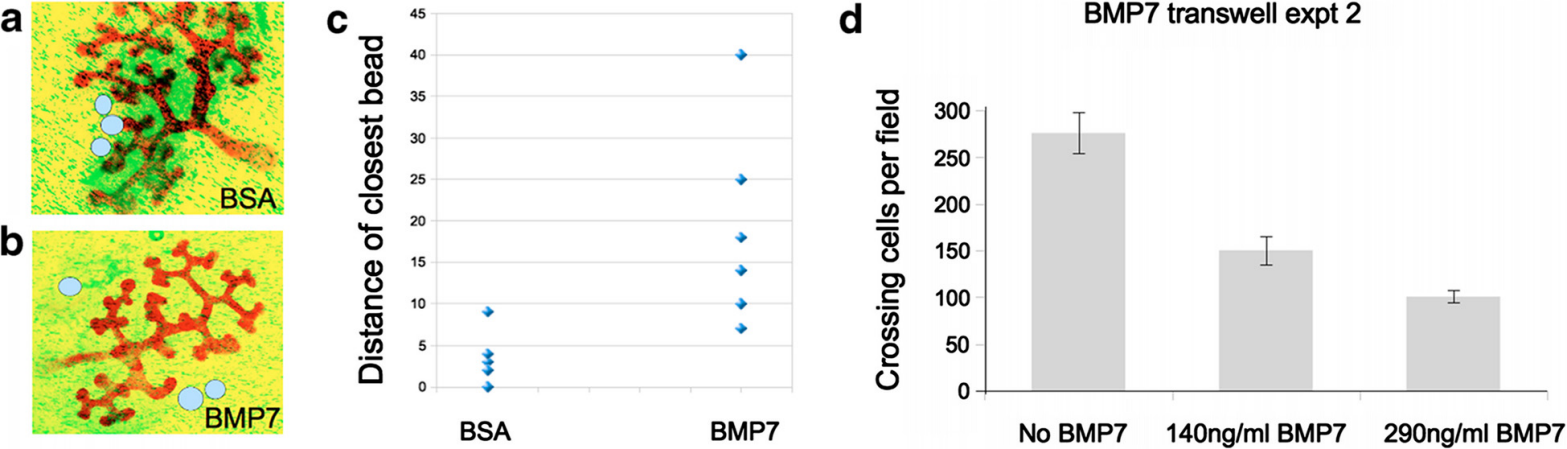
**Ureteric buds and their cells eschew sources of BMP7.** Placing control, BSA-soaked Affigel beads near ureteric buds **(a)** shows no obvious repulsive effect and branches will run into beads that happen to be in their way. In contrast, branches do not make contact with BMP7-soaked Affigel beads **(b)**, nor do they approach them very closely: these data are shown quantitatively in **(c)**, which shows the distance between the branch and bead that are closest in each culture. **(d)** In a completely different assay, ureteric bud-derived cells were cultured on filters, with medium supplemented with different concentrations of BMP7 under the filter. The graph shows the frequency of cells crossing the filter: filter crossing is inhibited by BMP7.

If BMP7 acts as an autocrine inhibitor, ureteric buds should avoid artificial sources of it. This was tested in two ways. In the first assay, Affigel beads soaked in either 100 ?g/ml BSA or in 100 ?g/ml BMP7, and blind-coded, were placed at the periphery of E11.5 kidney rudiments and the distance of closest approach of bud and bead in each culture was measured after 2¿3 days. Bud branches grew close to BSA-soaked beads, some making contact, although there was no evidence for attraction (Figure 8a). They remained significantly further away from BMP7-soaked beads, sometimes bending or remaining very short in their vicinity (Figure 8b). Quantitative analysis showed a significant difference in closest approach seen in each culture (Figure 8c; p?=?0.01). However, this assay suffers from unavoidable variability in the initial placement of beads so we used an alternative assay to confirm the effect of BMP7. We used a standard filter-crossing chemotaxis assay based on the 6TA2 immortalized ureteric bud cell line [11]. This assay works by seeding cells above an opaque filter and counting the number of cells to have crossed the filter towards a test medium after an interval of time. The logic of the assay requires the test medium and the medium above the cells not to have equilibrated before the end of the experiment. This was tested by a simple pilot experiment in which ink was added to one side of a cell-free filter, or as a control to the centre of a filter-free dish, and its progress into the compartment across the filter was assessed visually. With no filter present, the ink began to spread at once and reached equilibrium by 30 minutes; with the filter, the ink remained concentrated on one side of the filter even 28 hours later (photographs can be seen in Additional file 12: Figure S1). Diffusion through the filter pores is therefore too slow to destroy a concentration difference of even a small molecule, over the time-course of the real experiment. For the real experiment, 6TA2 cells were seeded above an opaque filter and the number of cells detectable below the filter was counted after 28 h (Figure 8d: this is a summary of experiments plotted separately in Additional file 13: Figure S2). The presence of BMP7 below the filter caused a significant, dose-dependent reduction of filter crossing to 64% of control values (p?=?6 × 10^?6^) towards 140 ng/ml BMP7 and to 29% of control values (p?=?9 × 10^?13^) towards 290 ng/ml. The simplicity of the cell line filter-crossing assay also indicates that ureteric bud cells are directly responsive to BMP7 in the absence of mesenchymal cells.

## Discussion and conclusion

The results presented above have shown that the ureteric bud system of kidney rudiments cultured flat show the wide first divergence angle and narrower subsequent angles that are typical of 3-dimensional organs in vivo [14]. Culture of tips from unbranched and once-branched buds showed that divergence angle is not controlled by different, sequential genetic programs but by proximity of other branches. A simple computer model suggested that this behaviour could be accounted for by a self-avoidance system, in which growing branches are repelled by something they themselves secrete. The implication of the model, that deliberate attempts to cause collisions between bud trees would be thwarted by self-avoidance, was confirmed by culture of real kidneys. Use of attempted collisions as an assay identified BMP signalling through Alk receptors as being critical to self-avoidance, with BMP7 being at least one of the molecules involved. Blocking BMP7 signalling produces a significant incidence of collisions, but many branches still appear to avoid contact, so there may in addition be parallel systems to keep them apart.

What does self-avoidance add to the existing repertoire of guidance systems, such as the biophysics of tube tips [25] and paracrine signalling from stroma [26],[27] believed to control pattern formation in tubule trees? First, self-avoidance can explain how branch divergence angles can change automatically from very open to more closed without any need for special, sequential systems. Instead, the changing anatomy may result from an unchanging mechanism of control. The system may therefore be simpler than it first appears. Second, self-avoidance might also provide a means of automatic error correction. A striking feature of organ culture, observed for many years although attention is not normally drawn to it, is that a ureteric bud tree that would normally grow and spread out three-dimensionally will, when cultured in a two-dimensional system, produce a tree that still spreads out without collisions. Simple reduction of the three-dimensional anatomy of a normal tree to two dimensions, for example in a projection, would produce an image in which many shadows of branches would cross. This is not what happens in culture: instead, buds adapt and produce a properly-spaced two-dimensional tree. This demonstrates both the flexibility of bud patterning and its ability to compensate for even large-scale departures from normal anatomy. Such compensation would be expected in a system using self-avoidance; proper spreading out of the branches of the two-dimensional trees in the computer model was driven by self-avoidance alone.

Self-avoidance is probably not critical to the formation of a tree in the first place. The BMP7^?/?^ mouse has severe renal dysgenesis with too few nephrons but it does have a small and cystic collecting duct system [28]. The presence of any kind of collecting duct system underlines the fact that an epithelial tree, albeit a morphologically-abnormal one, can be constructed even without BMP7-mediated self-avoidance. It is possible that other self-avoidance systems were still active; our data implicate BMP7 in self-avoidance but do not prove that it is solely responsible. It is also possible that other, cell-level mechanisms [26],[27] are enough to make a basic tree, and that self-avoidance is used only to mitigate the effects of occasional errors of positioning.

BMP7 is already known to be an inhibitor of the first emergence of the ureteric bud from the nephric duct [29] and, at high concentrations, an inhibitor of collecting duct cell line proliferation in culture [30]. Supporting this is the observation that the BMP receptor Alk3 is needed to prevent excessive ureteric bud branching [31]. BMP7 is not expressed in every organ that involves epithelial branching morphogenesis but organs may use different members of the TGF?-superfamily for the same purpose. The autocrine production of motility-inhibiting TGF? itself by mammary gland cells [13] suggests that mammary ducts may use a similar self-avoidance system, but based on TGF?. It may be that, just as different organs use different activators of branching (FGF7, FGF10, GDNF) that feed into similar intracellular pathways [32], so they use different members of the TGF?-superfamily as autocrine inhibitors. They may also use more than one molecule, just as many organs use more than one activator of branching. The main point of this report is not to argue for any particular molecule being generally important in self-avoidance, but is rather to illustrate that self-avoidance seems to exist, at least in kidney, and that it offers an explanation for branch angles changing during development and for branches not tangling even when the system is perturbed.

Inhibitory influences on the migrations of cells and cell processes are important in patterning other parts of the embryo, such as segmentation of the the peripheral nervous system [33], mapping of optic nerve to the colliculus in the brain [34], positioning of aortae each side of the midline [35], segmentation of intersomitic vessels [36], positioning the foregut and controlling the position at which the ureteric bud emerges from the Wolffian duct [37]. These systems use a variety of molecules, such as Ephs/Ephrins, Semaphorins, Robo/Slit and BMP4, sometimes balanced by their antagonists [34]¿[39]. There is evidence for repulsion being involved in the patterning of branching systems of bacterial colonies [40], dendritic trees [41] and fungal hyphae [42]. Some simple culture studies have suggested that epithelia derived from the branched tubes of mammary and salivary glands can show repulsion [13],[43]: here we have shown this repulsion at work in the context of a complete organ rudiment.

## Methods

### Kidney dissection and culture

Kidneys were dissected manually from E10.5 (unbranched UB for first-branch-angle experiments) and E11.5 (T-branched; used for all other experiments) CD-1 mouse embryos. For time-lapse images kidneys from intercrosses between Tg(Hoxb7-cre)13Amc/J [44] and Gt(ROSA)26Sor^tm1(EYFP)Cos^[45] were used. For tip angle experiments, ureteric bud tips were isolated manually, with the mesenchyme that stuck to them, using fine hypodermic needles. The bud tips were cultured on filters marked with a notch, to keep track of the orientation of the bud tip. For collision avoidance experiments, extraneous mesenchyme was removed from the rudiments, leaving only the bud and the dense mesenchyme surrounding it. This prevented cultured organs from becoming so thick that tubules could cross over/under one another without contact, giving a false impression of collision. The ureter itself was trimmed close to the kidney so that it did not interfere with potential tree-tree collisions. For collision avoidance experiments, two or more kidney rudiments were placed in direct contact: it was not possible to control their relative orientations so this was allowed to be random. Beads soaked in BMP7 or control proteins were placed at the periphery of kidney rudiments using pulled pasteur pipettes. The deep blue colour of the beads allowed them to be observed. Kidney rudiments grown for the time-lapse imaging were cultured on 0.4 ?m PET Transwell membranes (Corning), while all other rudiments were grown on fragments (about 5 mm × 5mm) of Millipore 0.4 ?m polycarbonate filters (Sigma P9449) supported at the air-medium interface on a stainless steel Trowell grid. In all cases, culture medium was Minimum Essential Eagle¿s with Earle¿s Salts (Sigma M5650) with 10% newborn calf serum and with penicillin and streptomycin (Sigma P5333 diluted 1/100 to the working concentration. When reagents were added, the appropriate equal volume of vehicle control was added to control cultures.

### Growth factors and inhibitors

Alk inhibitor II [2-(3-(6-Methylpyridin-2-yl)-1H-pyrazol-4-yl)1,5-naphthyrine] was obtained from Calbiochem (616452) and dissolved at 5 mg/ml in DMSO. Gremlin was from R&D systems (956-GR), reconstituted to 250 ?g/ml in 4 mM HCl with 0.1% bovine serum albumin, as recommended by the manufacturer. BMP7 was from R&D systems (5666-BP) and reconstituted to 100 ?g/ml in 4 mM HCl with 0.1% bovine serum albumin, again as recommended by the manufacturer: polyclonal anti-BMP7 was Aviva (ARP32329).

### Immunostaining, imaging and quantification

Cultured kidney rudiments were fixed in ?20°C methanol, which was allowed to warm towards room temperature over 15 minutes and then replaced by phosphate buffered saline (PBS). Still attached to their filters, they were stained overnight in 1/100 mouse anti-calbindinD^28k^ (abcam 82812) and, in some cases, 1/200 rabbit anti-Six2 (LSBio LS-C10189), washed for 6-8 h in PBS, incubated overnight in FITC anti-mouse (Sigma F2012) for most experiments, and TRITC anti-mouse (Sigma T5393) and FITC anti-rabbit (Sigma F0382) for the Six2 staining experiments, and washed for 2-4 h in PBS. After staining, samples were mounted in 50% PBS: 50% glycerol, between 22 × 64mm coverslips, themselves separated by 22 × 22mm coverslips at their ends to maintain a space for the samples. The coverslip sandwich was then placed on a microscope slide for observation using a Zeiss epifluorescence microscope (the coverslip sandwich technique was used so that it could be turned over if the filter happened to be mounted kidney side-down).

Branch angles were measured manually, by electronically drawing skeleton lines along the centre of ureteric bud trunk and branches then measuring the divergence angles with a protractor on a printout of each image. For collision avoidance experiments, collisions were defined as approaches so close that no gap could be discerned by light microscopy: cultures were scored categorically, as having collisions or not having them. For bead experiments, measurements of closest approach were made by measuring the distance between the nearest edges of the bead and the branch that were the closest in each culture. Measurements of tip growth velocities in time-lapse movies were made by examining successive frames. For measuring the speed of free tips (for Figure 5b), the x and y pixel coordinates in frame n and frame n?+?1, taken 1 h apart, were recorded, and the distance travelled was calculated as ?[(x_n+1_-x_n_)^2^?+?(y_n+1_-y_n_)^2^]: this was done every 5 frames. Speed was calculated as difference in location divided by elapsed time. For approaching tips (for Figure 5c), the x and y pixel coordinates of each of two nearby tips were recorded in frame n and frame n?+?1, the distance between the two tips was calculated (Pythagoras) in frame n and frame n?+?1, and approach velocity was recorded as the difference between the distance at frame n?+?1 and at frame n, divided by elapsed time. Between four and ten tip pairs were recorded in this way per frame (early frames include few nearby tips, later frames include more because there are more tips in all by then). This analysis was performed using LibreOffice Calc.

### Exclusion criteria

For the angle experiments in Figure 2, all samples were included. For collision avoidance experiments, only cultures that had no gap between the kidneys were included in the analysis.

### Computer modelling

Modelling was done using the *Processing* language: a description of the model, and its source code, appear separately in the Supplementary Data (Additional file 1: Code S1, Additional file 2: Movie S1, Additional file 3: Movie S2a, Additional file 4: Movie S2b, Additional file 5: Movie S3, Additional file 6: Movie S4, Additional file 7: Spreadsheet S1, Additional file 8: Text S1 and Additional file 9: Text S2). For simulations of collision experiments, a variety of anatomical starting conditions was used to correspond with what was done in real culture. A selection of these conditions is available in the program (see program notes in Supplementary Material (Additional file 1: Code S1, Additional file 2: Movie S1, Additional file 3: Movie S2a, Additional file 4: Movie S2b, Additional file 5: Movie S3, Additional file 6: Movie S4, Additional file 7: Spreadsheet S1, Additional file 8: Text S1 and Additional file 9: Text S2)).

### Chemotaxis assays

For cell line-based chemotaxis assays, 6TA2 ureteric bud cells [46] were seeded on the top surface of BD Falcon¿ FluoroBlok¿ Cell Culture Inserts for 24-well plates, 8.0 ?m (cat. 351152, BD Biosciences) and pre-incubated for 24 h with medium both above and below the inserts. The culture medium medium consisted of DMEM-F12 (Sigma D8437) with 10% FCS (Invitrogen 10108165), 1x ITS (insulin, transferrin, selenium) supplement (Sigma. I3146), 1x antioxidant supplement (Sigma A1345), and 1x penicillin-streptomycin-glutamine mix (Invitrogen 10378016). BMP7 (0, 140 or 290 ng/ml) was then added to the lower solution, the cells were incubated for a further 4 h, then the filters were removed, fixed in 4%PFA for 20 min, washed in 0.1% Triton X-100 (cat. H0934, Sigma) in 1X PBS (cat. P4417, Sigma) for 5 min, stained with propidium iodide (cat. P3566, Molecular Probes) and FITC Phalloidin (cat. P5282, Sigma) washed in 0.1% Triton X-100 in 1X PBS for 10 min mounted inverted and the number of cells spreading out from filter pores per microscope field was counted (images being blind-coded). Only fields in which the filter edge did not encroach were counted. For preliminary diffusion experiments using ink, a drop of Parker Quink fountain pen ink was placed in the centre of either a 3 cm petri dish containing 3mls PBS, of in the contained space of a Fluoroblok cell culture insert in a similar 3 cm petri dish containing 3mls PBS: the fluoroblok cell culture insert was filled with PBS to the same level as the surrounding dish. Photographs were taken using a hand-help camera at intervals from 0-4 h.

### Statistical calculations

For continuously-variable quantitative data, standard deviations and standard errors of the mean were used to indicate variation and t-tests were used for testing significance. For scoring proportions of cultures showing collisions (each individual culture yielding a `categorical¿ yes/no state rather than a continuously-variable quantity), 95% confidence intervals were calculated as ±1.96?(p(1-p)/n)?+?1/2n [47]. Hypothesis testing for these data was performed using two sample *z* tests [48]. For analysis of the relationship between velocity and log of proximity in time-lapse movies (Figure 5c), linear regression was applied, using the `LINEST¿ function built into the LibreOffice Calc spreadsheet software.

### Ethical statement

This work involved no human material or data, and involved no experiments on living animals. Tissues were obtained from animals killed by a method approved by Schedule 1 of the UK Animals (Scientific Procedures) Act, by licenced technical staff in Home-Office licenced premises.

## Competing interests

The authors declare that they have no competing interests.

## Authors¿ contributions

JAD designed the experiments, performed all of the renal cultures except the time-lapse imaging, analysed all organ culture results including that of the movie and wrote the simulation software. PH and RB produced the time-lapse images. C-HC performed the cell line-based taxis assays. All authors read and approved the final manuscript.

## Additional files

## Supplementary Material

Additional file 1: **Code S1.** Simulation code. Click here for file

Additional file 2: **Movie S1.** Simulation of a single ureteric bud, guided by self-avoidance. Click here for file

Additional file 3: **Movie S2a.** Simulation of two ureteric buds growing directly at one another. Click here for file

Additional file 4: **Movie S2b.** Simulation of two ureteric buds growing directly at one another. Click here for file

Additional file 5: **Movie S3.** Simulation of two ureteric buds approaching one another obliquely. Click here for file

Additional file 6: **Movie S4.** A Hoxb7-cre x ROSA-eYFP kidney rudiment growing in culture. Click here for file

Additional file 7: **Spreadsheet S1.** Anxalysis of movies of real kidney growth. Click here for file

Additional file 8: **Text S1.** Index of supplementary files. Click here for file

Additional file 9: **Text S2.** A brief explanation of the model. Click here for file

Additional file 10: **Figure S4e.** Analysis of network topology in Figure S4e. Click here for file

Additional file 11: **Figure S4f.** False-colour version of Figure S4f in the main paper. Click here for file

Additional file 12: **Figure S1.** Pilot transfilter diffusion experiments using ink. Click here for file

Additional file 13: Transfilter assessment of 6TA2 ureteric bud cell migration. Click here for file
