# Supplementary figures and images for: A self-avoidance mechanism in patterning of the urinary collecting duct tree
Source: BMC Dev Biol. 2014 Sep 10;14:35. doi: 10.1186/s12861-014-0035-8 (PMC4448276; doi:10.1186/s12861-014-0035-8)

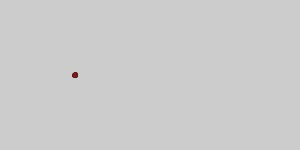

Supplement: Additional file 2: — Movie S1. Simulation of a single ureteric bud, guided by self-avoidance. [file s12861-014-0035-8-S2.gif]

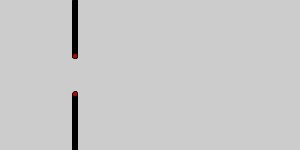

Supplement: Additional file 3: — Movie S2a. Simulation of two ureteric buds growing directly at one another. [file s12861-014-0035-8-S3.gif]

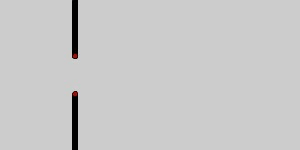

Supplement: Additional file 4: — Movie S2b. Simulation of two ureteric buds growing directly at one another. [file s12861-014-0035-8-S4.gif]

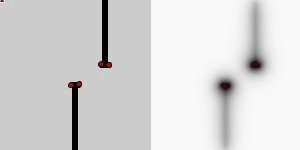

Supplement: Additional file 5: — Movie S3. Simulation of two ureteric buds approaching one another obliquely. [file s12861-014-0035-8-S5.gif]

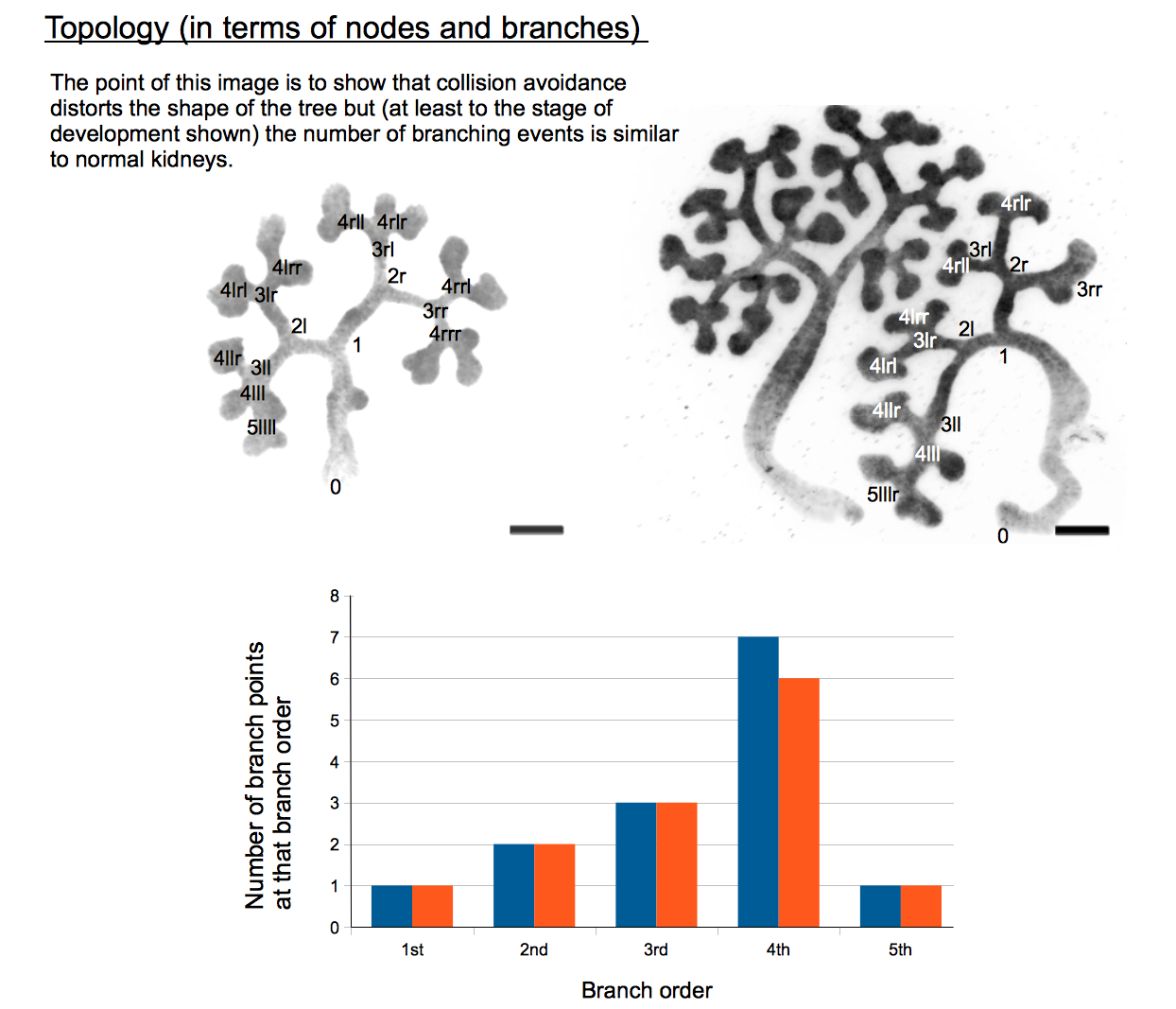

Supplement: Additional file 10: — Figure S4e. Analysis of network topology in Figure S4e. [file s12861-014-0035-8-S10.tiff]

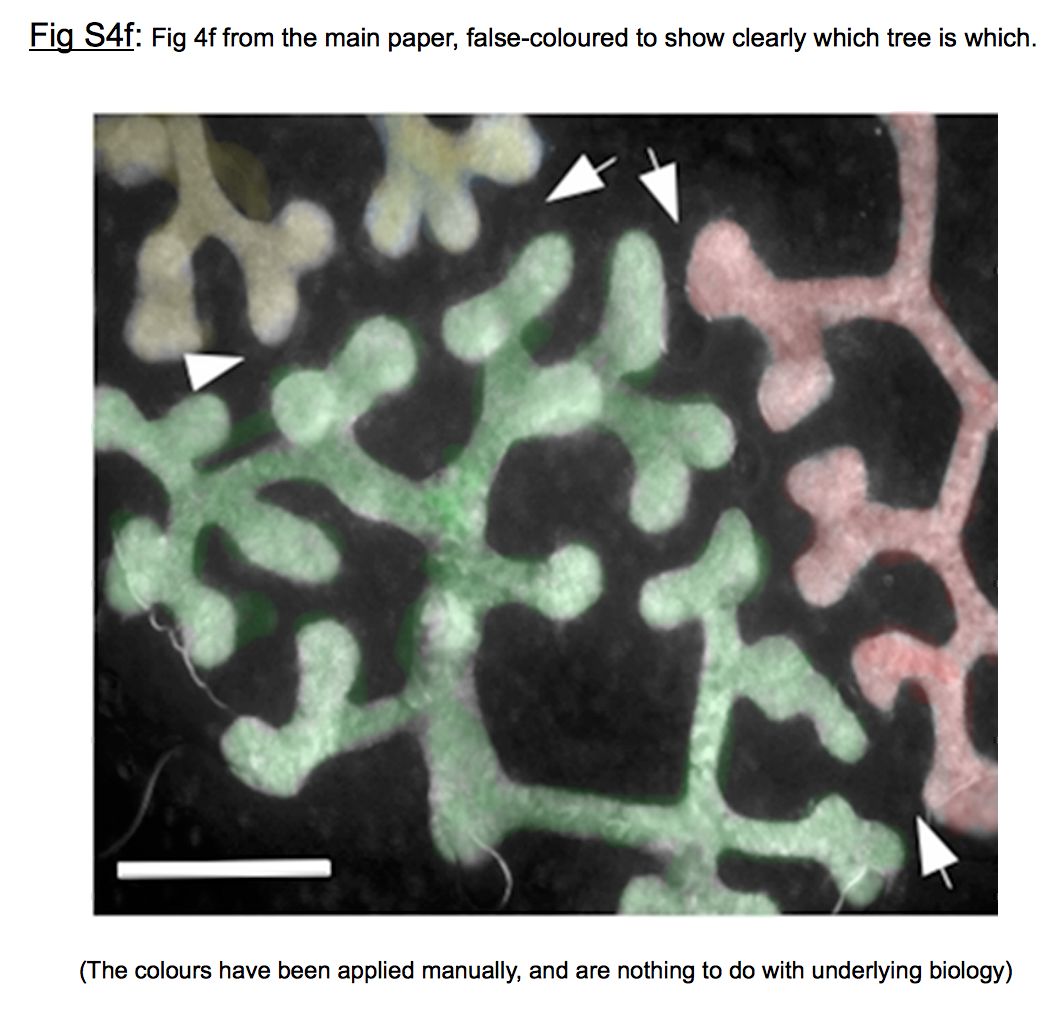

Supplement: Additional file 11: — Figure S4f. False-colour version of Figure S4f in the main paper. [file s12861-014-0035-8-S11.tiff]

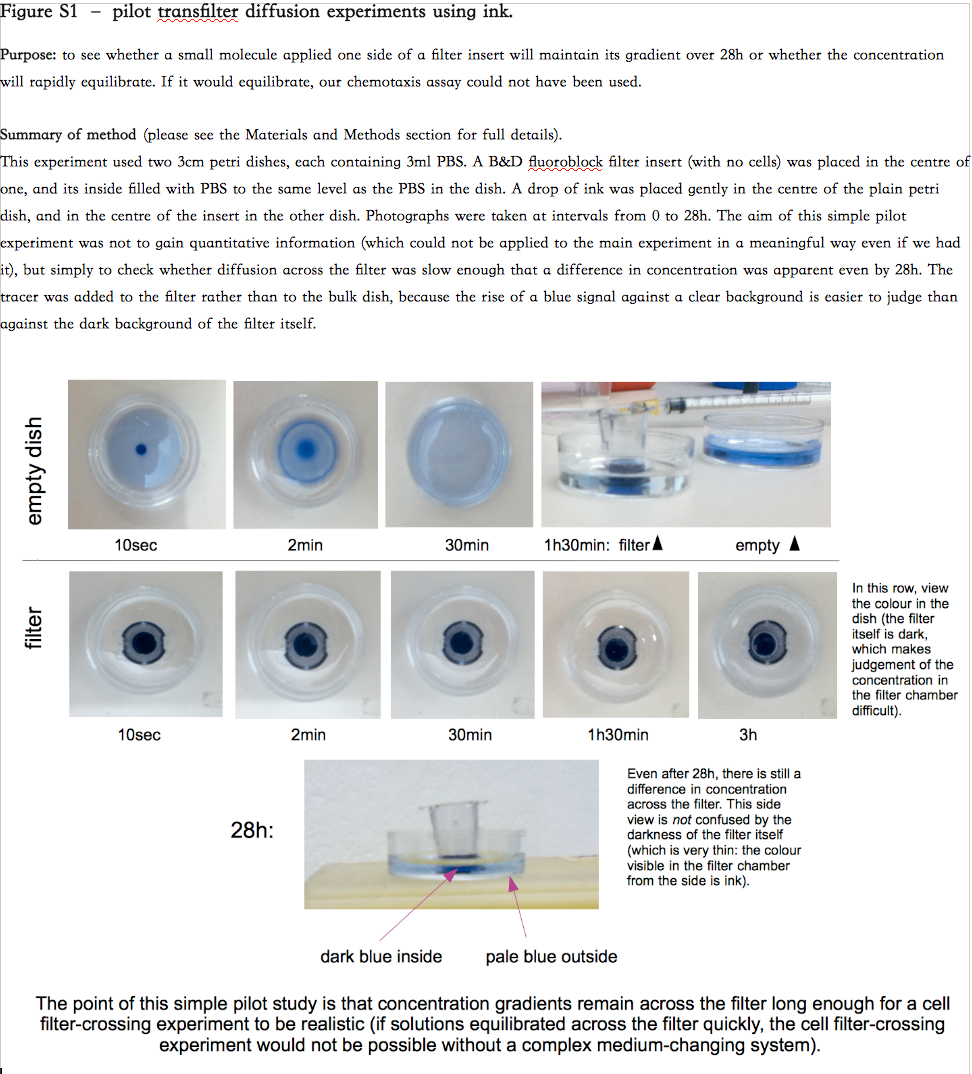

Supplement: Additional file 12: — Figure S1. Pilot transfilter diffusion experiments using ink. [file s12861-014-0035-8-S12.tif]

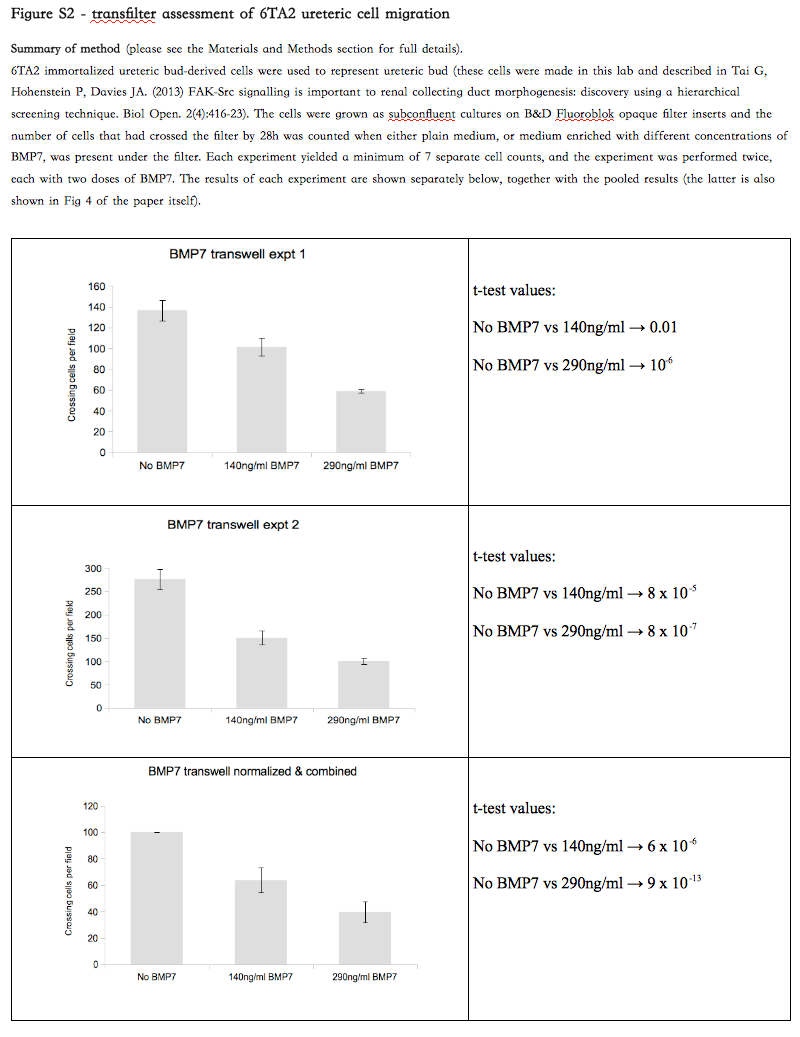

Supplement: Additional file 13: — Transfilter assessment of 6TA2 ureteric bud cell migration. [file s12861-014-0035-8-S13.tif]
