# Supplementary material for: A self-avoidance mechanism in patterning of the urinary collecting duct tree
Source: BMC Dev Biol. 2014 Sep 10;14:35. doi: 10.1186/s12861-014-0035-8 (PMC4448276; doi:10.1186/s12861-014-0035-8)
Supplement: Additional file 8: — Text S1. Index of supplementary files. [file s12861-014-0035-8-S8.pdf]

Text S1: Index of Supplementary Files

Example movies of computer simulations

- Movie S1: Simulation of a single ureteric bud, guided by self-avoidance
- Movie S2a: Simulation of two ureteric buds growing directly at one another
- Movie S2b: Simulation of two ureteric buds growing directly at one another
- Movie S3: Simulation of two ureteric buds approaching one another obliquely.

Code

- Text S2: a brief explanation of the model
- Code S1: simulation code

Example movie of real kidney growth

- Movie S4: a Hoxb7-cre x ROSA-eYFP kidney rudiment growing in culture
- Spreadsheet S1: analysis of movies of real kidney growth

Figures of experimental data

- Figure S1: Transfilter gradient pilot experiments with ink.
- Figure S2: BMP7 experiments plotted separately (with p values).
- Figure S4e: Analysis of network topology in Fig 4e
- Figure S4f: False-colour version of Fig 4f in the main paper.
