# Supplementary material for: A self-avoidance mechanism in patterning of the urinary collecting duct tree
Source: BMC Dev Biol. 2014 Sep 10;14:35. doi: 10.1186/s12861-014-0035-8 (PMC4448276; doi:10.1186/s12861-014-0035-8)
Supplement: Additional file 9: — Text S2. A brief explanation of the model. [file s12861-014-0035-8-S9.pdf]

Text S2: A brief guide to the model of self-avoidance

Contents:

1. Biological aspects of the model
2. Computational aspects of the model

Biological aspects

The model supposes that ureteric bud secretes a molecule (called 'horrid' in the model: a name chosen deliberately because it is not already the name of a known protein). Both stalk and tip make it, and the model allows the amount made by each to be specified so that they can make the same or different amounts, including zero. These are the rules of the model:

- Tips and stalks make and secrete horrid

- Concentrations of horrid fall exponentially with distance from their source (this is an assumption of 1st order decay: we have no reason to assume any particular kinetics, so chose simplest and most common). The model assumes that molecular turnover and diffusion are so fast compared to anatomical change (roughly 1 new branch order per day in real kidneys) that the concentration field can be calculated from current anatomy, with no need for memory of past states. This avoids the need for guessing about a lot of unknown kinetic constants. The concentration of inhibitor I at position x,y is therefore calculated as

$$I_{x,y} = \sum_{i,j} \text{sourceconc} \cdot \exp\left[-\text{decay} / \sqrt{\{(i-x)^2 + (j-y)^2\}} \right]$$

  where i,j are the coordinates of all points in the centre-line of the ureteric bud system, and sourceconc is, for each i,j, appropriate to whether the i,j location is a stalk or a tip.

- Tips advance quickly with low [horrid] and slow down as [horrid] increases, with a linear relationship until they stop completely at a critical threshold (*stallthreshold*). Since advance is in practice quantized (pixels), this is realized by using a probability of advancing one pixel, the probability being set by

$$p = (\text{stallthreshold} - [\text{horrid}]) / \text{stallthreshold}$$

  (unless this expression would yield a negative number, in which case p=0).

cont...

- Tips advance in the direction of lowest horrid concentration (as measured at the tip surface). Where two or more directions have equal concentrations, the tip chooses between them

randomly.

- All tips bifurcate when the local concentration of horrid is below a threshold (*branchthreshold*).
- Stalk elongation behind branch points is not modelled, as it would be an irrelevant complication (though it does happen in real life later in development, when the medullary ducts elongate: these events are beyond the scope of this study).

Computational aspects.

This program is written for maximum 'show your working' clarity so that its operation can be easily understood and modified. It therefore avoids tricks that could improve speed and efficiency at the expense of obfuscation. Also, it was written to be played with by scientists who understand it, not to be an idiot-proof toy for random button pushing. Biological parameters are loaded at the top of the code, and are altered by editing that code (they cannot be altered while the program is running). I have indicated limits to variables next to where they are defined at the start of the program. If clear instructions such as 'this value must be divisible by 3' (eg for a variable called 'fieldsize', which sets up the simulation space) are ignored then silly things will happen or the program will halt with an error. I have assumed that this program will be used and altered only by someone competent to do so.

The system is modelled on a 2D grid, the dimensions of which are set by 'fieldsize'. There are 2 such grids, one 'actual' one representing state at time *t* and one a temporary one in which the state at *t+1* is calculated. The actual is updated only after the entire temporary field has been calculated. Each location of the fieldsize grid is used to contain a concentration of 'horrid'. It *also* contains anatomical information because the code 888888 is used to indicate that a location corresponds to the centreline of a stalk, and the code 999999 is used to indicate that a location corresponds to the centre of a tip. These numbers are not mistaken for horrid concentrations because the program detects them and replaces them, in drawing out the horrid field, with the correct values to be found in a stalk or tip. The technique is used to save having to have a whole new array for the anatomy. The starting anatomy is setup by a called-once-only function: different starting points can be created by re-writing this function (and a choice of pre-prepared anatomies is available in the code as it stands).

The main loop ('draw') calls functions to calculate the concentration of horrid across the field according to current anatomy, to add some noise, to update the anatomy by advancing each tip in a

direction that involves least horrid (see below), to draw out the new horrid field and anatomy, to save a movie frame if the movieflag is set, and then begins again. It is the nature of this programming language that 'draw' runs forever unless stopped manually.

Calculation of the horrid field is done location-by-location with no attempt at short-cuts. Each time, concentrations are calculated de novo based on the distance of that location from every tip and stalk. For each field location, the program scans the whole of the field and, when it finds a tip or stalk (indicated by the 999999 and 888888 codes respectively) it calculates the distance from that location and adds an amount of horrid based on exponential decay over that distance. Contributions from each tip and stalk are summed. Biologically speaking, this means that we assume that diffusion and destruction of molecules is fast compared with tissue growth, so that history is of marginal relevance and concentrations can be predicted from current anatomy.

Calculation of the direction of tip growth is a little complicated in order to avoid a problem that besets many computational models of morphogenesis: this problem is an artefact of square arrays that results in everything seeming to lie at some exact multiple of 45 degrees. In this program, for each tip, the program centres itself in the tip, 'walks' round the tip in 0.1 radian steps, and records the angle *phi* at which the amount of horrid at the tip surface is least. As tips advance by just one location (pixel) at a time, there are just 8 choices of direction in which the next advance can be made (up, up right, right.. etc). The program identifies the two neighbouring pixel directions between which the true angle *phi* lies. It then makes a choice of moving to one pixel or the other based on a probability calculation such that if *phi* lies exactly between 2 available pixel directions, each has an equal chance of being chosen while if it lies closer to one, then that one's being chosen is more probable. The actual probability distribution is based on a cosine curve.

If there is low enough horrid for a tip to branch, the original tip is deleted and two new ones are created, separated by a short distance (variable *newtipstep* – set so that new tips appear immediately adjacent on the anatomy drawing), at right angles to the existing stalk (ie the new tips are side-by-side from the perspective of the stalk). A short length of new stalk is added as a 'T'-bar across the old stalk to connect the new tips.

Tips are not allowed to get closer than 1 pixel to the edge of the field (they just stop advancing).

Making Movies

If *movieflag* is set to 1, successive frames will be dumped in the same directory as the program (before running it, flush out any image files from previous runs or you risk making movies that have frames from different simulation). You can turn the set of frames into an animated gif using ImageJ. Use File->Import->Image sequence, click on the first image, and let ImageJ import: it will by default import all subsequent frames in the directory.

Use File->Save as-> Animated GIF to save.

Animated GIFs will play in Firefox (and other things) and can be mounted directly on the web.
